# Supplementary material for: Clinical Phenotypes of Severe Cow’s Milk Protein Allergy with Various Responses to Amino Acid-Based Formula
Source: Nutrients. 2025 May 26;17(11):1809. doi: 10.3390/nu17111809 (PMC12158079; doi:10.3390/nu17111809)
Supplement: Supplementary file 1 [file nutrients-17-01809-s001.zip › nutrients-3621999-supplementary.pdf]

Supplementary materials

Table S1. Additional diagnostic procedures.

Variable	n (% of group)
Clinical assessment	232 (100)
Elimination diet	198 (85.3)
Family interview	177 (76.3)
sIgE assessment/food panel/component resolved diagnostics	53 (22.8)
Oral provocation test at home	26 (11.2)
Oral provocation test at doctor's office	6 (2.6)
Atopic patch test	4 (1.7)
Skin prick test	2 (0.9)
Oral provocation test in clinical center	1 (0.4)
Other*	4 (1.7)

* Including: general feces examination, dermatological consultation, bacteriological feces examination, SARS COV19 tests.

Table S2. Baseline symptoms severity by cluster (n = 229)

Symptoms	Cluster A (GI) n = 90	Cluster B (skin) n = 79	Cluster C (GI + skin) n = 60	p	post hoc test *
All symptoms **	17.12 ± 8.11	12.52 ± 4.76	26.02 ± 7.44	< 0.001[1]	AB, AC, BC[2]
All symptoms > 0 on visit I **	17.12 ± 7.82	12.49 ± 4.73	25.90 ± 7.25	< 0.001[1]	AB, AC, BC[2]
**Skin**					
Erythema	0.31 ± 0.68	2.05 ± 0.89	2.17 ± 0.69	< 0.001	AB, AC
Itching	0.28 ± 0.56	2.09 ± 0.98	2.22 ± 0.69	< 0.001	AB, AC
Dry skin	0.71 ± 0.97	2.41 ± 0.81	2.53 ± 0.68	< 0.001	AB, AC
Rush/eczema	0.50 ± 0.80	2.22 ± 0.92	2.42 ± 0.70	< 0.001	AB, AC
Angioedema	0.01 ± 0.11	0.13 ± 0.37	0.10 ± 0.30	0.019	AB
Urticaria	0.17 ± 0.55	0.33 ± 0.65	0.27 ± 0.61	0.113	-
**Gastrointestinal**					
Stomach ache	1.82 ± 1.08	0.34 ± 0.70	2.03 ± 0.84	< 0.001	AB, BC
Diarrhea	1.72 ± 1.12	0.20 ± 0.52	1.83 ± 0.85	< 0.001	AB, BC
Nausea	0.22 ± 0.58	0.00 ± 0.00	0.17 ± 0.38	0.001	AB, BC
Vomiting	0.67 ± 0.97	0.06 ± 0.29	0.67 ± 0.82	< 0.001	AB, BC
Regurgitation	1.77 ± 1.06	0.43 ± 0.84	2.08 ± 0.83	< 0.001	AB, BC
Burping	1.10 ± 1.11	0.05 ± 0.27	1.23 ± 1.03	< 0.001	AB, BC
Bloating	1.96 ± 1.03	0.29 ± 0.62	2.20 ± 0.73	< 0.001	AB, BC
Decreased appetite	1.07 ± 1.16	0.06 ± 0.25	1.02 ± 1.08	< 0.001	AB, BC
Blood/mucus in feces	1.33 ± 1.13	0.14 ± 0.45	1.40 ± 0.91	< 0.001	AB, BC
Intestinal colic	1.74 ± 1.04	0.25 ± 0.54	2.08 ± 0.91	< 0.001	AB, BC
Constipation	0.46 ± 0.88	0.20 ± 0.72	0.35 ± 0.71	0.016	AB
FPIES	0.14 ± 0.49	0.01 ± 0.11	0.08 ± 0.33	0.059	-
Other	0.04 ± 0.33	0.00 ± 0.00	0.03 ± 0.18	0.304	-
**Respiratory**					
Runny nose	0.07 ± 0.29	0.16 ± 0.52	0.23 ± 0.50	0.025	AC
Restricted nasal passage	0.14 ± 0.46	0.28 ± 0.66	0.42 ± 0.81	0.067	-
Chronic cough	0.10 ± 0.40	0.03 ± 0.16	0.05 ± 0.22	0.438	-
Wheezing breath	0.14 ± 0.55	0.14 ± 0.47	0.20 ± 0.58	0.724	-
Laryngeal edema	0.03 ± 0.23	0.03 ± 0.23	0.02 ± 0.13	0.895	-
Dyspnea	0.11 ± 0.46	0.15 ± 0.53	0.02 ± 0.13	0.201	-
Other					
**Others symptoms**					
Tearing eyes	0.00 ± 0.00	0.01 ± 0.11	0.00 ± 0.00	0.387	-
Itching eyes	0.02 ± 0.21	0.01 ± 0.11	0.00 ± 0.00	0.387	-
Eye redness	0.00 ± 0.00	0.04 ± 0.25	0.00 ± 0.00	0.697	-
Anxiety	0.16 ± 0.62	0.11 ± 0.45	0.07 ± 0.36	0.149	-
Sleep disorders	0.09 ± 0.47	0.10 ± 0.41	0.05 ± 0.29	0.651	-
Apathy	0.02 ± 0.21	0.03 ± 0.16	0.03 ± 0.26	0.704	-
Paleness	0.03 ± 0.32	0.08 ± 0.42	0.00 ± 0.00	0.790	-
Heavy sweating after a meal	0.02 ± 0.21	0.00 ± 0.00	0.00 ± 0.00	0.205	-
Growing disorders	0.16 ± 0.65	0.08 ± 0.42	0.05 ± 0.29	0.462	-
Other food allergy	0.00 ± 0.00	0.01 ± 0.11	0.00 ± 0.00	0.744	-

SD – standard deviation, Severity of symptoms could range from 0 (symptom not present) to 3 (very high intensity). Significance of difference between groups verified with Kruskal-Wallis test or Welch Anova analysis[1].
* Significantly different pairs of clusters, based on post-hoc Dunn test with Bonferroni adjustment or Tukey test[2].
** “All symptoms” variable was an aggregation of intensity of all symptoms and could range from 0 to 105.
FPIES, food protein-induced enterocolitis syndrome; GI, gastrointestinal.

Table S3. Demographic and clinical characteristics of the separate cohort (validation group) (n = 157)

Variable	n (% of group)
**Demographic data**	
Female	67 (42.7)
Male	90 (57.3)
Age, days (mean±SD)	129.77 ±100.9
Age, months (mean±SD)	4.25 ± 3.4
Height - percentile*	
< 3	9 (5.7)
3-10	18 (11.5)
10-25	33 (21.0)
25-50	33 (21.0)
50-75	35 (22.3)
75-90	20 (12.8)
90-97	6 (3.8)
> 97	3 (1.9)
Weight - percentile *	
< 3	2 (1.3)
3-10	10 (6.4)
10-25	35 (22.3)
25-50	31 (19.7)
50-75	43 (27.4)
75-90	24 (15.3)
90-97	8 (5.1)
> 97	4 (2.5)
Allergic diseases in family	80 (50.9)
**Clinical data**	
General severity	
mild	40 (25.5)
moderate	90 (57.3)
severe	27 (17.2)
Skin symptoms only	93 (59.2)
mild	30 (19.1)
moderate	50 (31.8)
severe	13 (8.3)
Gastrointestinal (GI) symptoms only	5 (3.2)
mild	2 (1.3)
moderate	3 (1.9)
severe	0 (0.0)
GI and skin symptoms	59 (37.6)
mild	7 (4.5)
moderate	34 (21.6)
severe	18 (11.5)

M – mean, SD – standard deviation
* Percentiles based on WHO growth references for part of patients and *Polish Institute of Mother and Child* growth references for others.

Table S4. Age, height and weight by cluster (n = 229)

Variable	Cluster A (GI) n = 90	Cluster B (skin) n = 79	Cluster C (GI + skin) n = 60	p	Post hoc test ***
Age, days*	128.12 ± 85.31	160.48 ± 80.39	137.60 ± 79.15	0.038[1]	AB[2]
Height, cm**	62.80 ± 8.45	65.49 ± 5.87	63.68 ± 5.72	0.003	AB
Weight, kg	6.15 ± 2.10	7.01 ± 1.91	6.50 ± 1.60	0.003	AB

SD – standard deviation, Significance of difference between groups verified with Anova analysis[1] and Kruskal-Wallis test.
* Based on n = 230 due to missing data for two patients.
** Based on n = 231 230 due to missing data for one patient.
*** Significantly different pairs of clusters, based on post-hoc Tukey test[2] and Dunn test with Bonferroni adjustment.
GI, gastrointestinal.

Figure S1

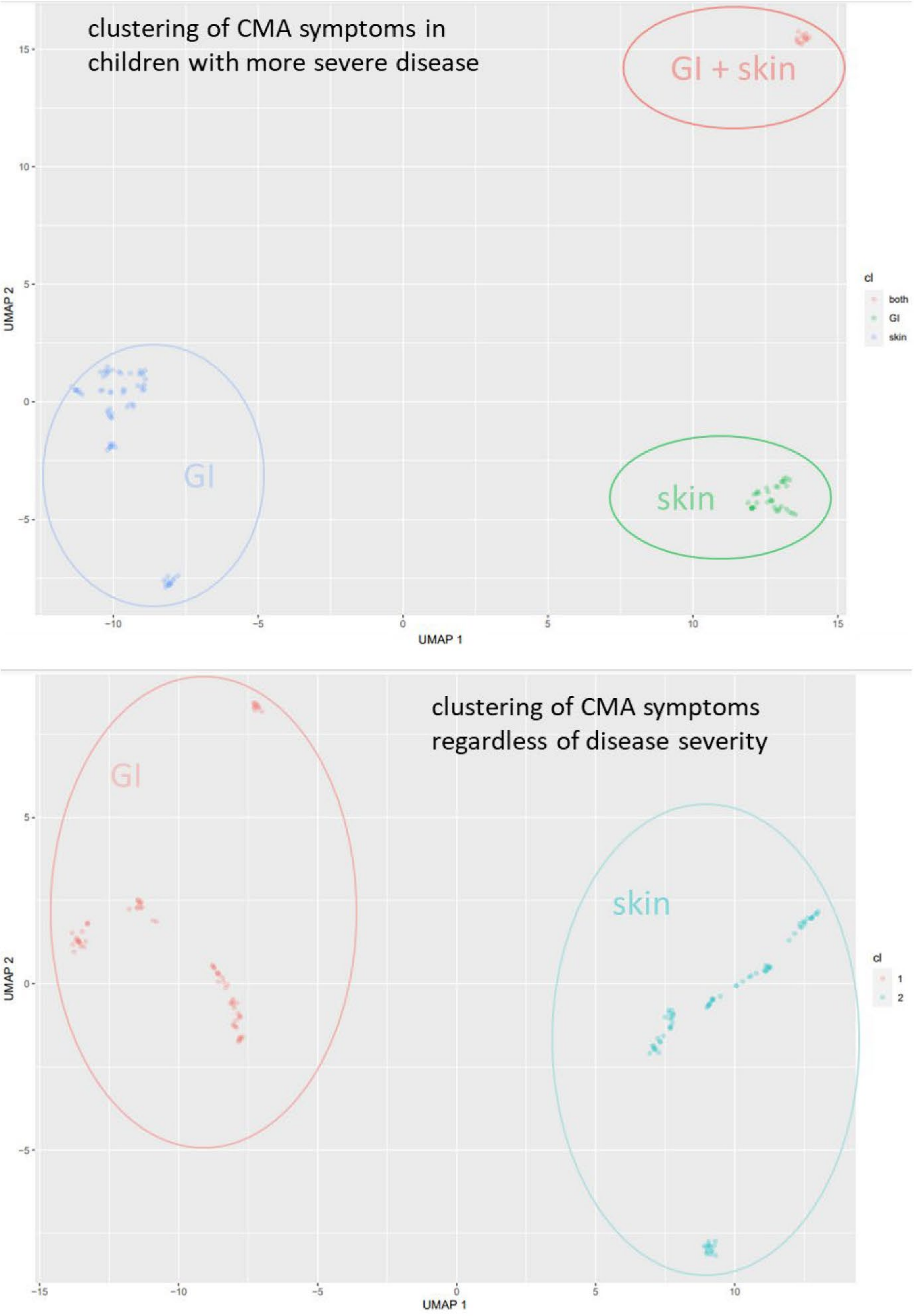
clustering of CMA symptoms in
children with more severe disease
GI + skin
GI
skin
UMAP 2
UMAP 1
cl
both
GI
skin
clustering of CMA symptoms
regardless of disease severity
GI
skin
UMAP 2
UMAP 1
cl
1
2
